# Supplementary material for: Routine measurement of cardiometabolic disease risk factors in primary care in England before, during, and after the COVID-19 pandemic: A population-based cohort study
Source: PLoS Med. 2024 Nov 26;21(11):e1004485. doi: 10.1371/journal.pmed.1004485 (PMC11593757; doi:10.1371/journal.pmed.1004485)
Supplement: S1 File — Fig A. Flowchart of individual exclusions. Fig B. Monthly number of measurements per 1,000 individuals by age group. Fig C. Monthly number of measurements per 1,000 individuals by sex. Fig D. Monthly number of measurements per 1,000 individuals by ethnicity. Fig E. Monthly number of measurements per 1,000 individuals by IMD quintile. Fig F. Estimated % deviation in number of measurements by sex. Fig G. Estimated % deviation in number of measurements by ethnicity. Fig H. Estimated % deviation in number of measurements by IMD quintile. (PDF) [file pmed.1004485.s002.pdf]

Supplementary Figures

Contents

Fig A. Flowchart of individual exclusions 2
Fig B. Monthly number of measurements per 1,000 individuals by age group 3
Fig C. Monthly number of measurements per 1,000 individuals by sex 4
Fig D. Monthly number of measurements per 1,000 individuals by ethnicity.......................... 5
Fig E. Monthly number of measurements per 1,000 individuals by IMD quintile 6
Fig F. Estimated % deviation in number of measurements by sex 7
Fig G. Estimated % deviation in number of measurements by ethnicity................................ 8
Fig H. Estimated % deviation in number of measurements by IMD quintile 9

Fig A. Flowchart of individual exclusions

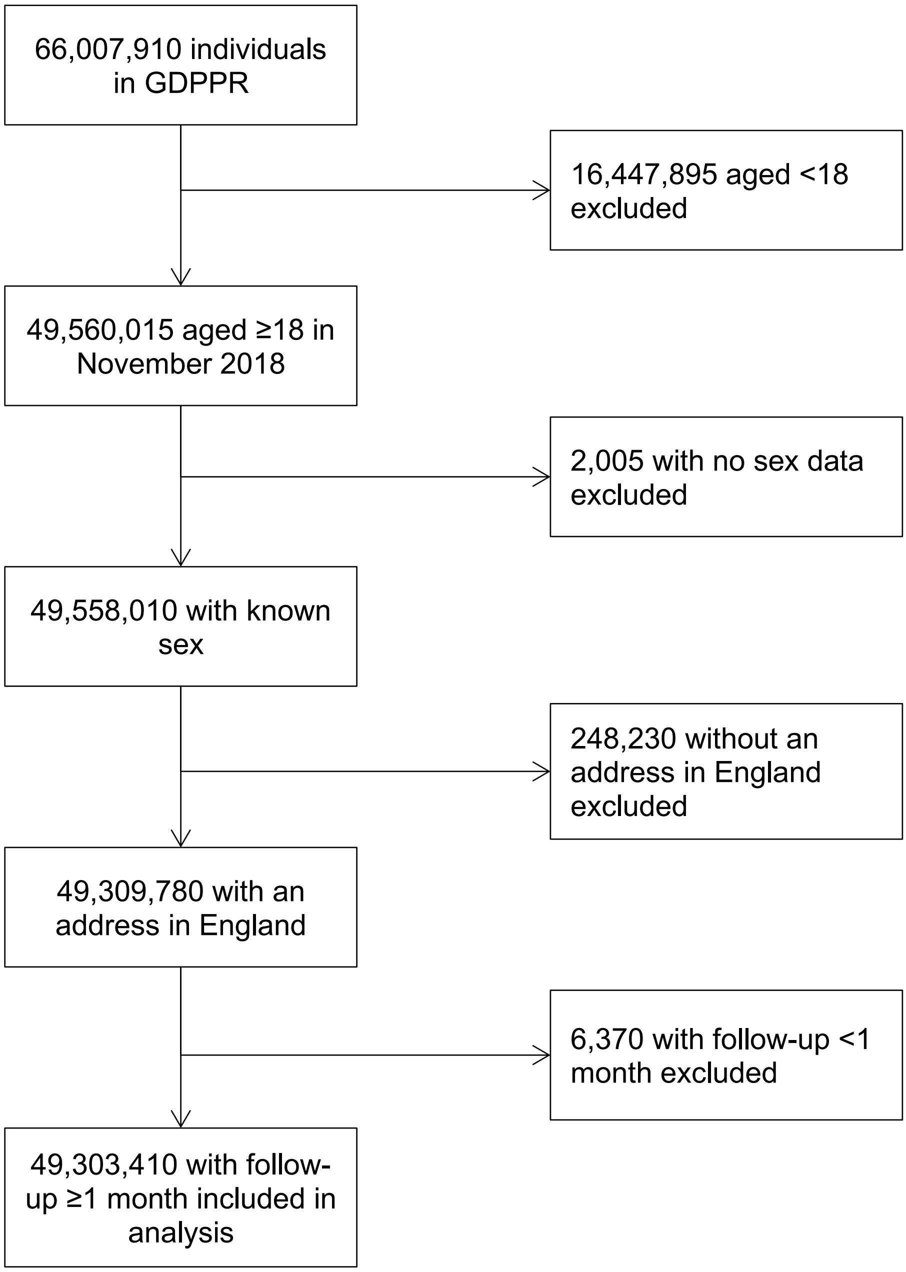

Fig B. Monthly number of measurements per 1,000 individuals by age group

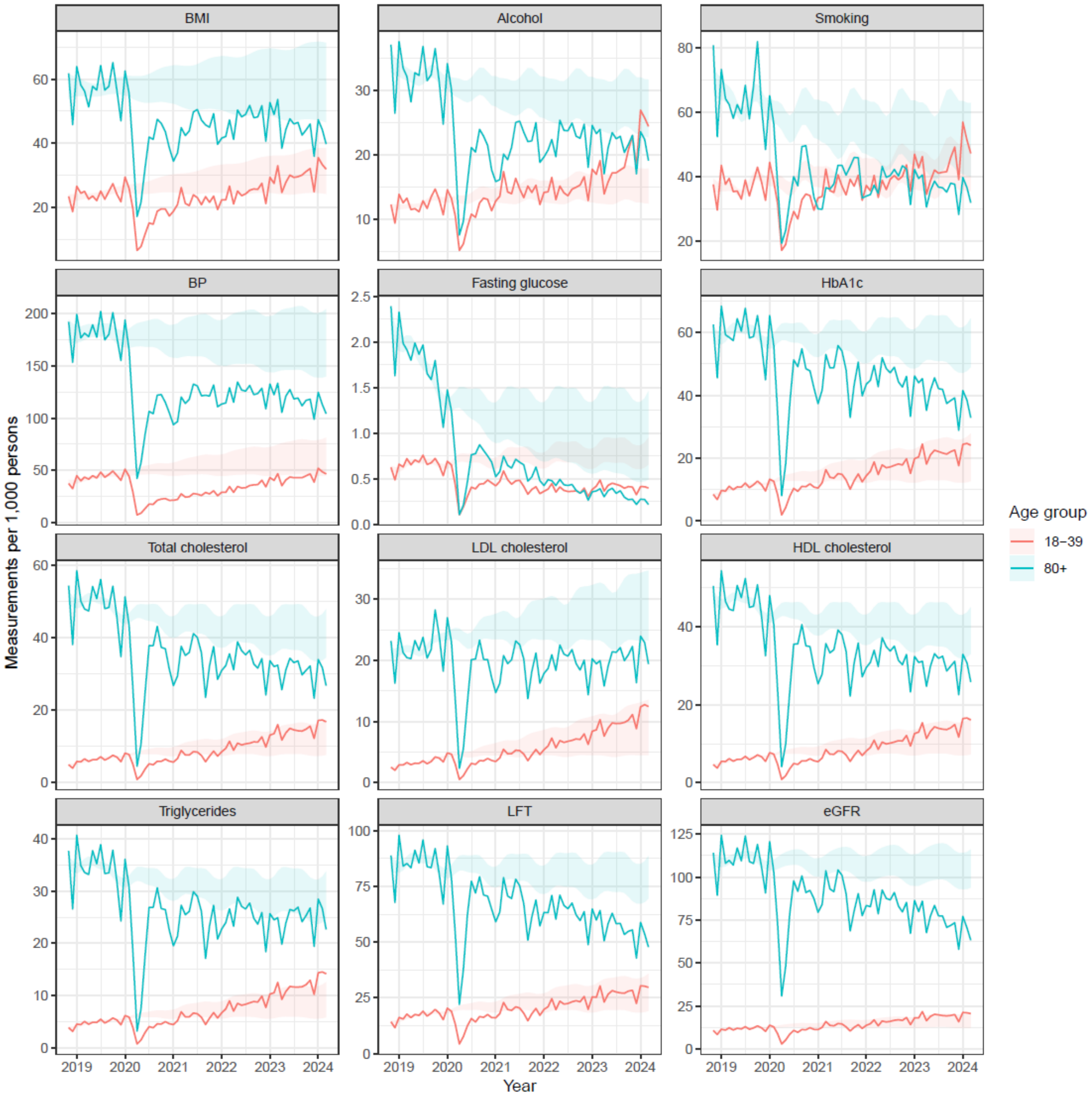

Shaded areas are ranges of expected numbers, aggregated from projected trends based on data between November 2018 and February 2020, and assumed stable trends from February 2020.

Fig C. Monthly number of measurements per 1,000 individuals by sex

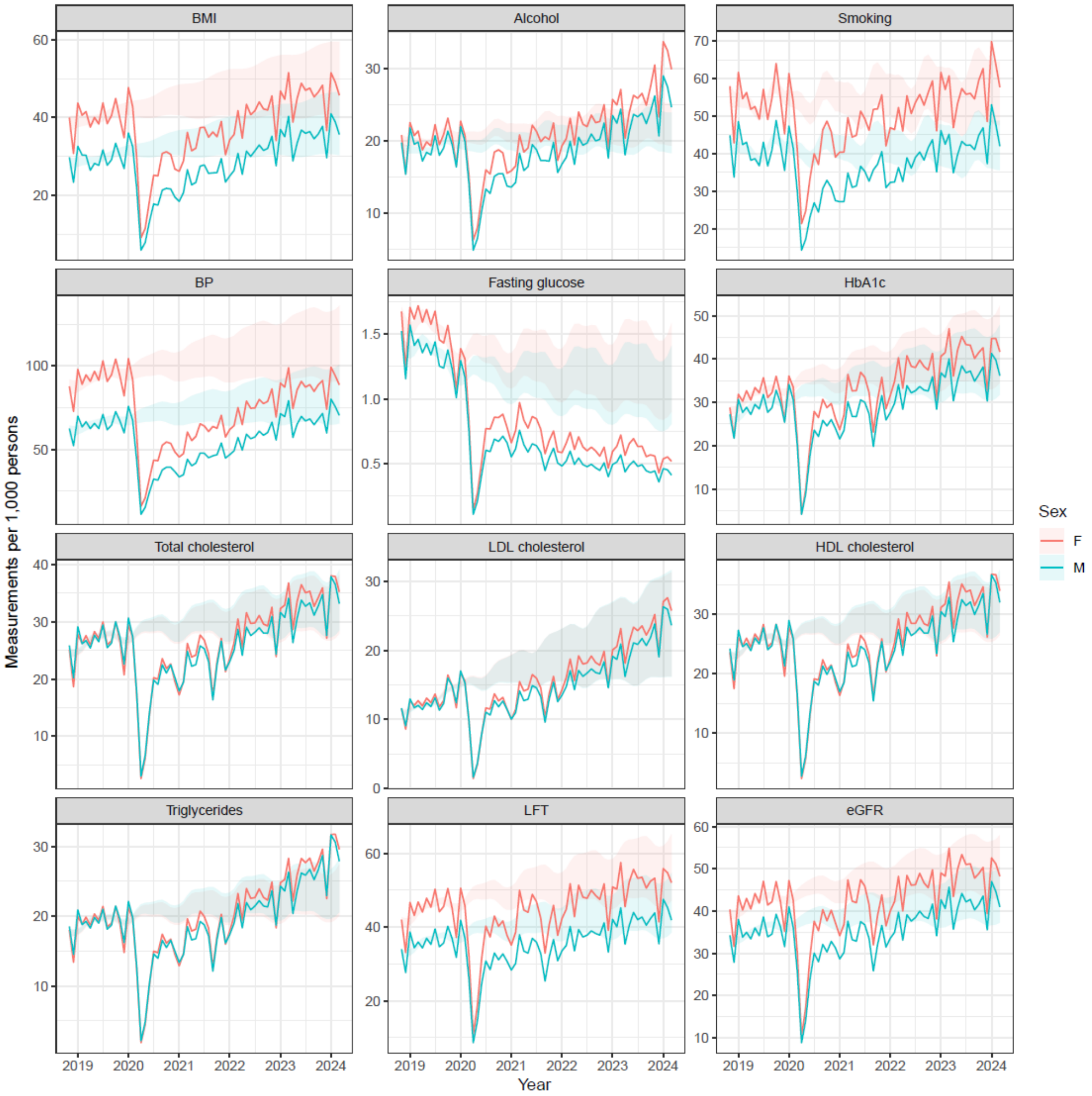

Shaded areas are ranges of expected numbers, aggregated from projected trends based on data between November 2018 and February 2020, and assumed stable trends from February 2020.

Fig D. Monthly number of measurements per 1,000 individuals by ethnicity

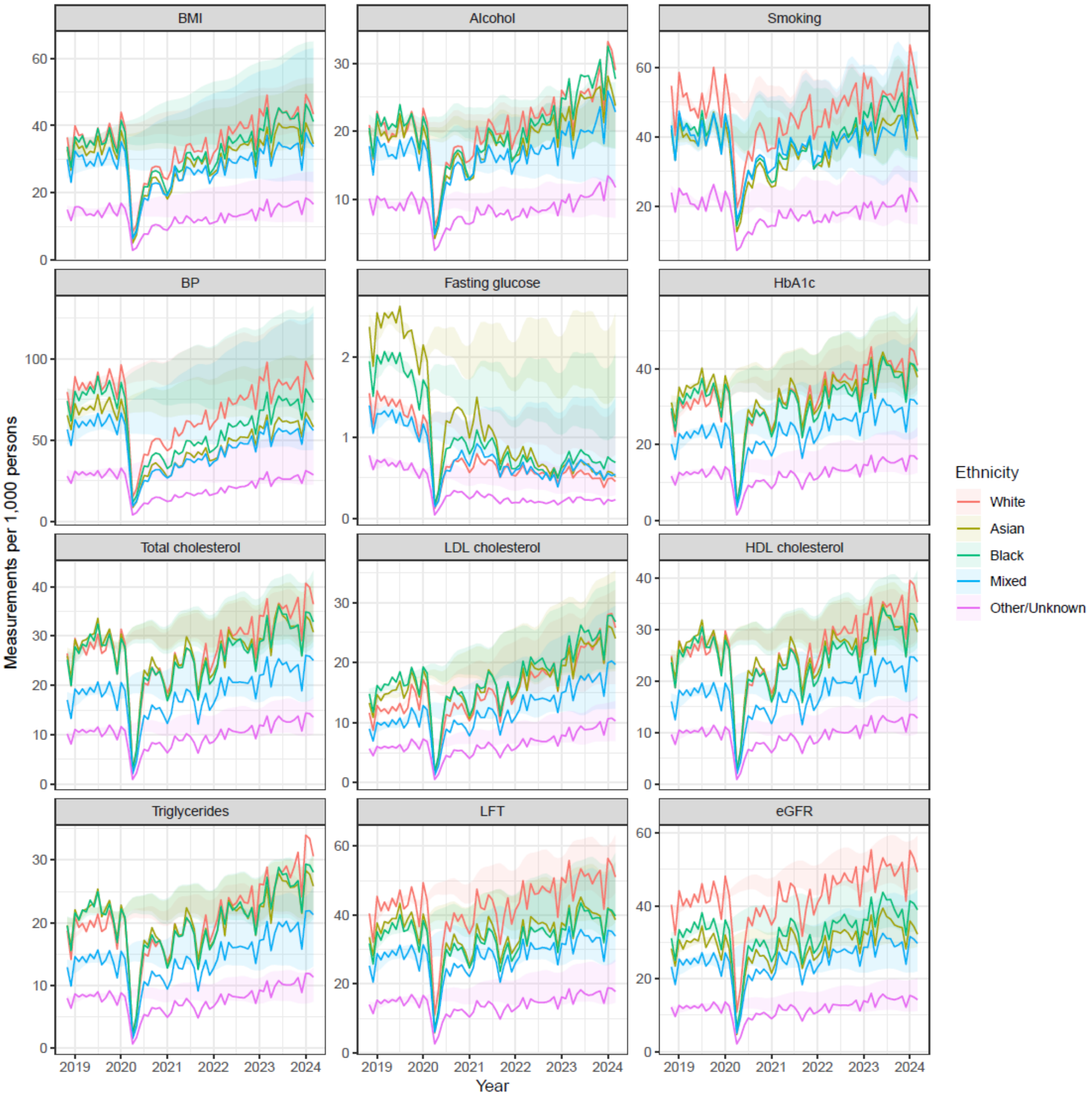

Shaded areas are ranges of expected numbers, aggregated from projected trends based on data between November 2018 and February 2020, and assumed stable trends from February 2020.

Fig E. Monthly number of measurements per 1,000 individuals by IMD quintile

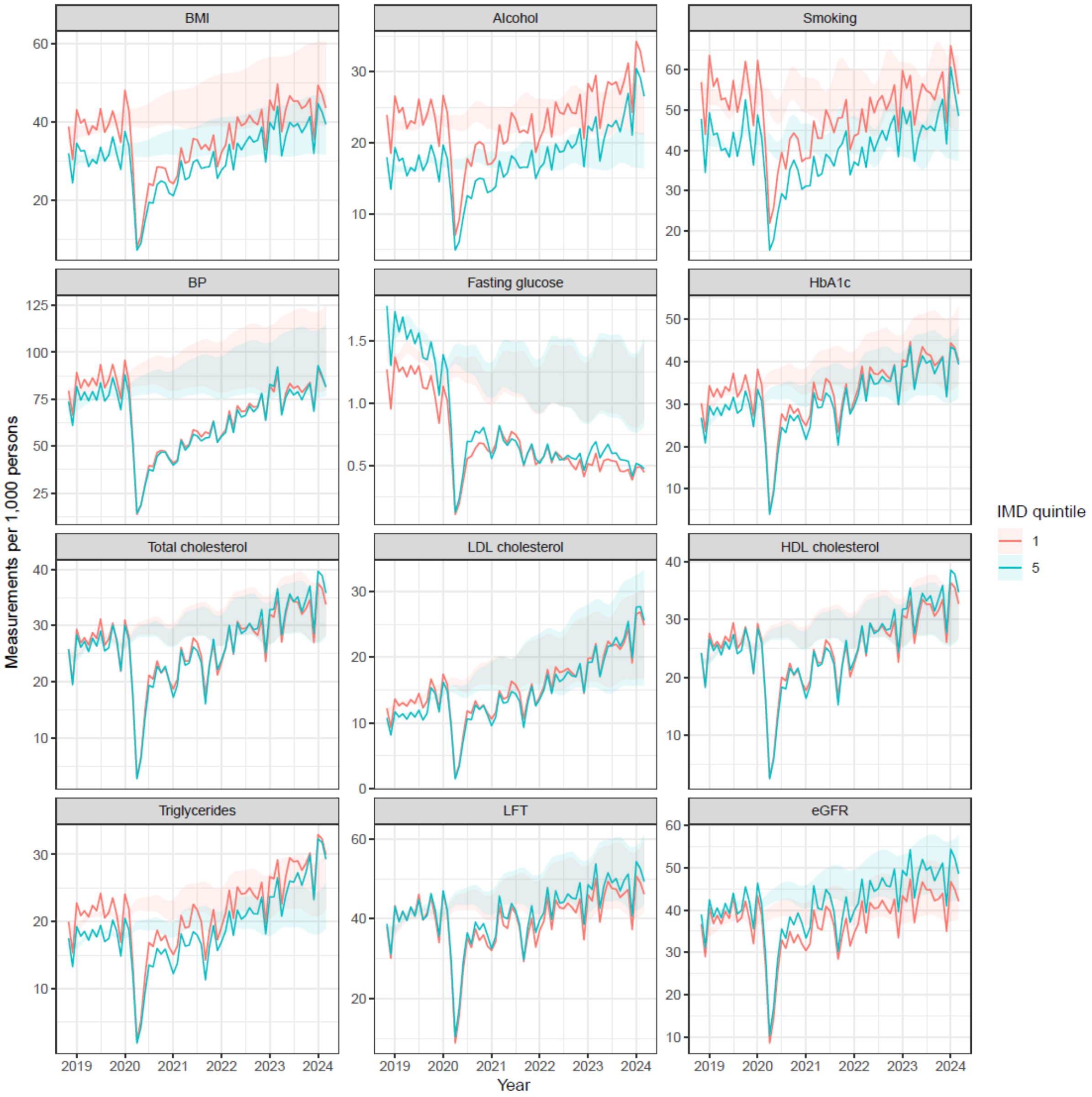

Shaded areas are ranges of expected numbers, aggregated from projected trends based on data between November 2018 and February 2020, and assumed stable trends from February 2020.

Fig F. Estimated % deviation in number of measurements by sex

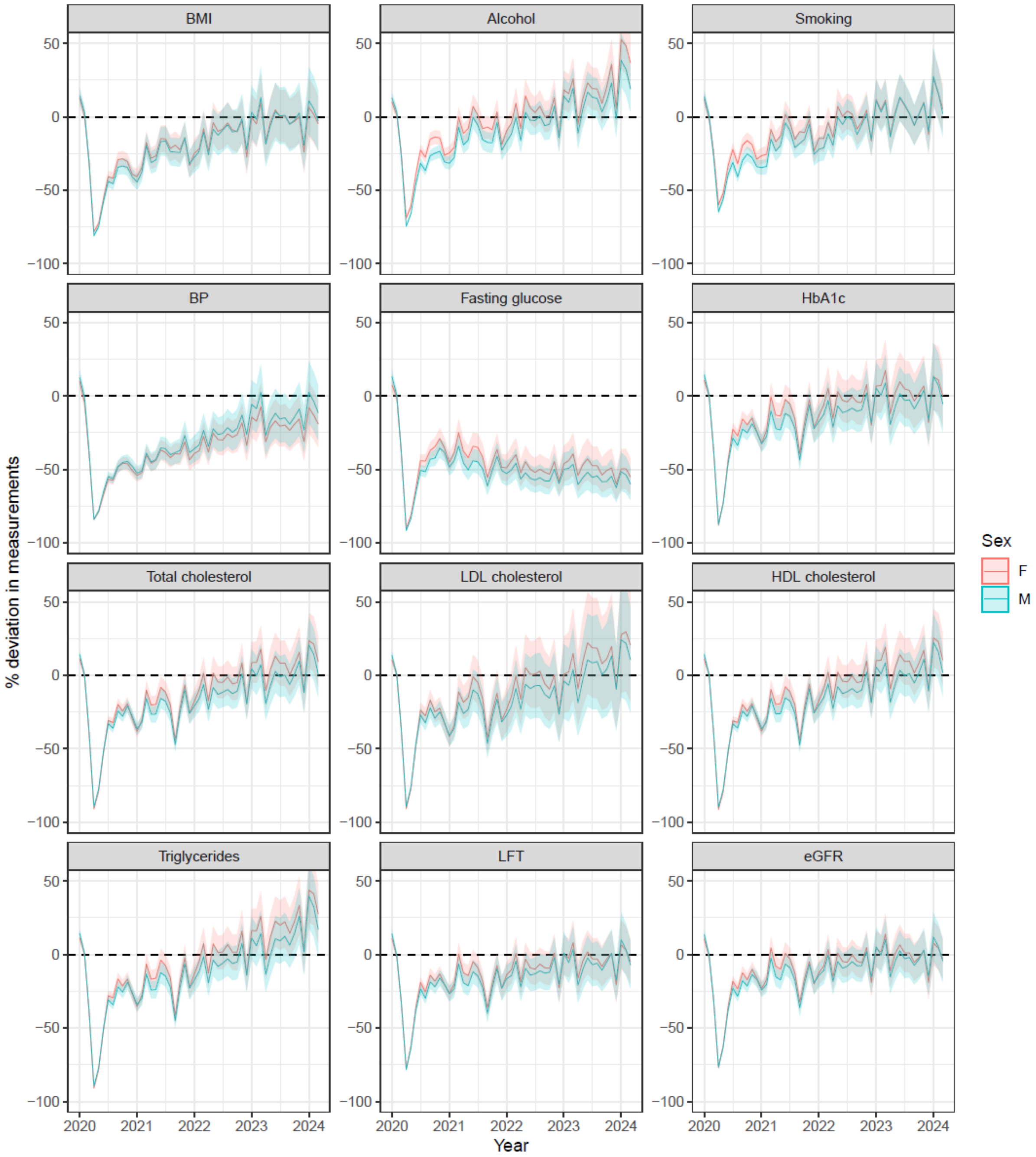

% deviation was estimated as observed - expected / expected.

Fig G. Estimated % deviation in number of measurements by ethnicity

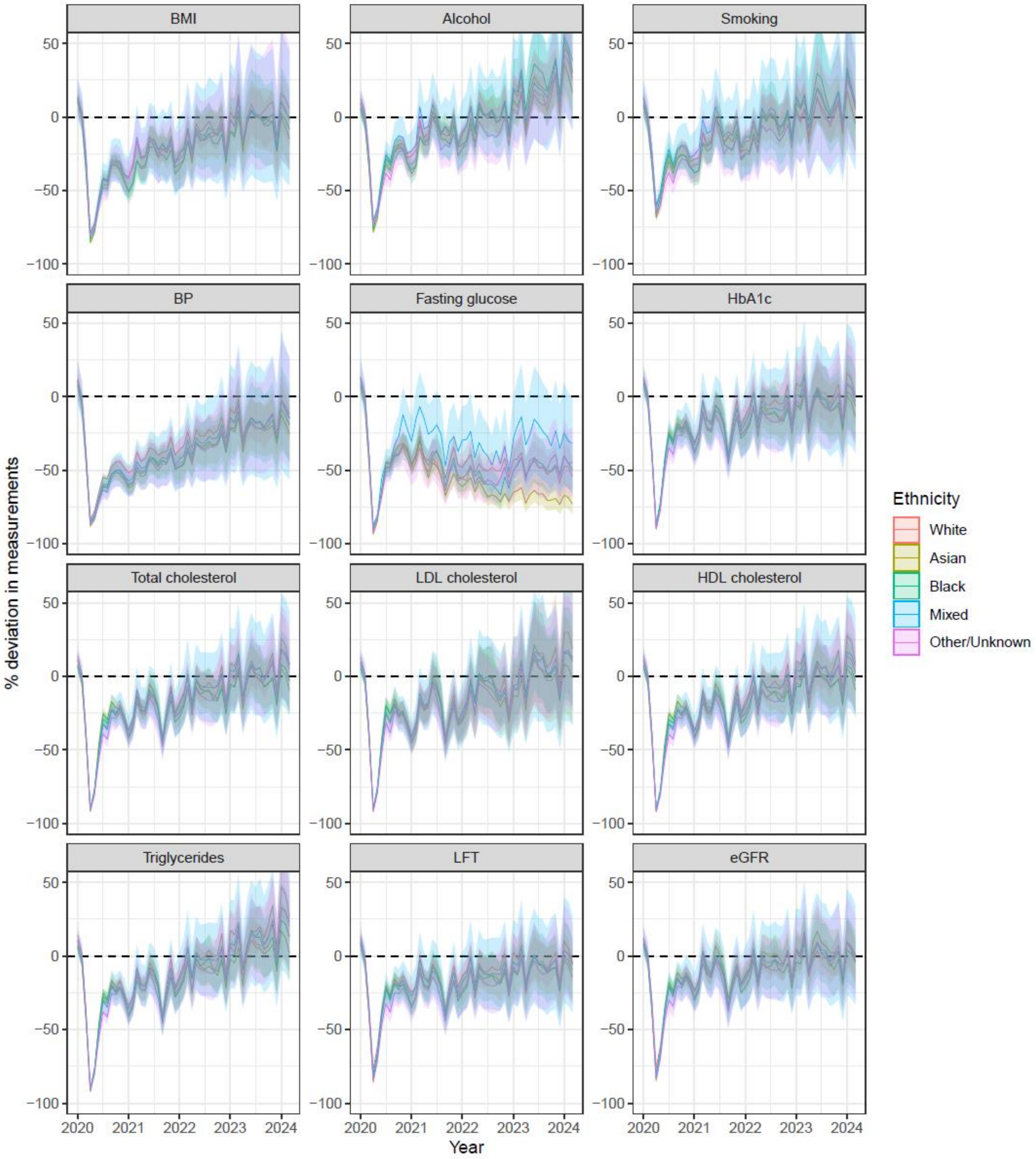

% deviation was estimated as observed - expected / expected.

Fig H. Estimated % deviation in number of measurements by IMD quintile

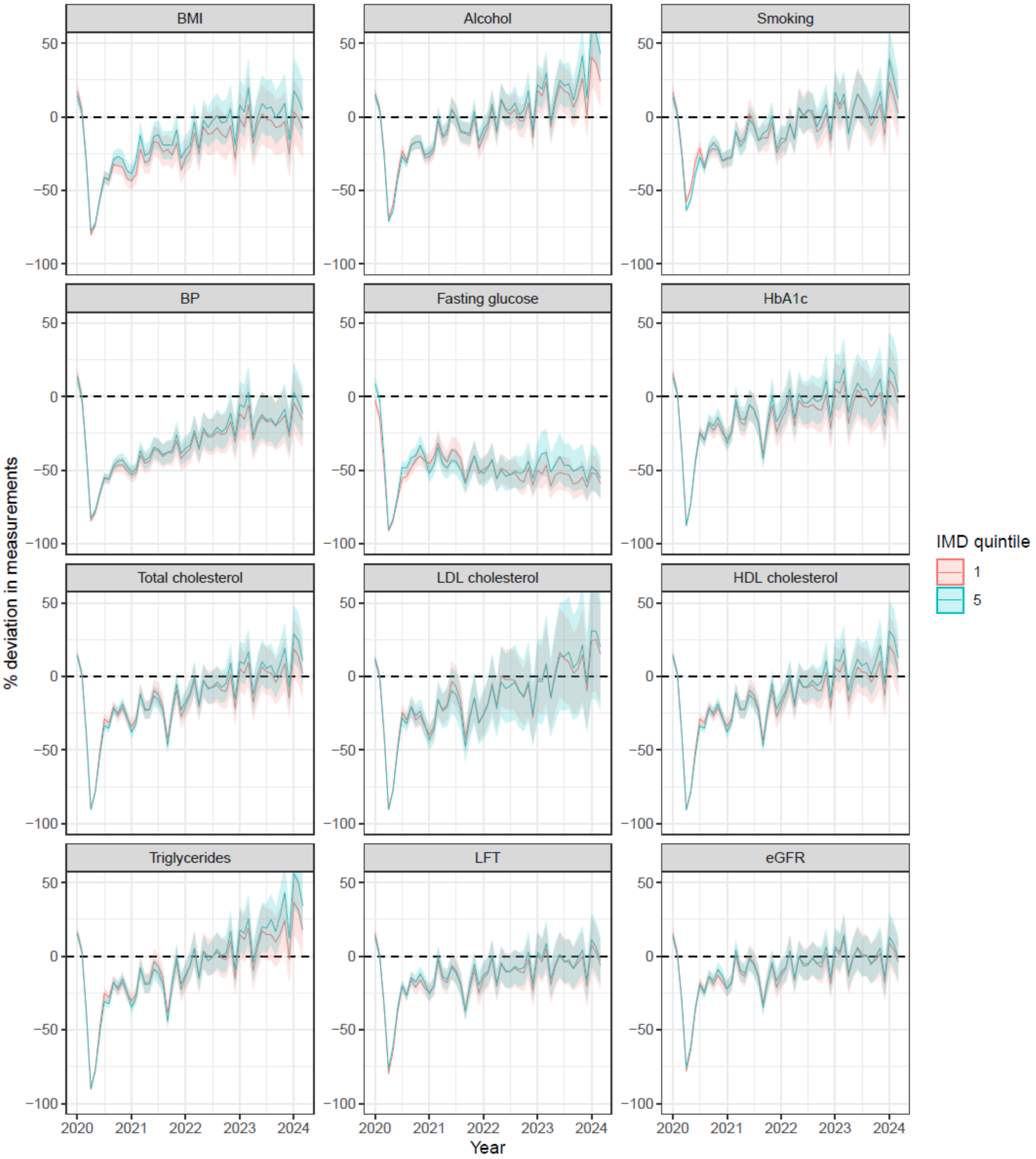

% deviation was estimated as observed - expected / expected.
