# Supplementary material for: Routine measurement of cardiometabolic disease risk factors in primary care in England before, during, and after the COVID-19 pandemic: A population-based cohort study
Source: PLoS Med. 2024 Nov 26;21(11):e1004485. doi: 10.1371/journal.pmed.1004485 (PMC11593757; doi:10.1371/journal.pmed.1004485)
Supplement: Attachment — Submitted filename: CCU008_01 number of risk factor paper r1v2 - Responses.docx [file pmed.1004485.s004.docx]

Editorial comments:

1) We liked your paper and agree with the reviewers that it offers a helpful addition to the existing data regarding the impact of the pandemic and the trajectory of the post-pandemic recovery of health services in the UK. As currently presented it is not without limitations, as identified by the reviewers, which we require you address prior to further consideration.

RESPONSE: Thank you for the appreciation. We have made substantial re-analyses address the reviewers’ excellent comments, including:

1. A defined cohort and focusing on number of measurements per individuals to account for competing risk.
2. Updated data (available until March 2024)
3. Included ethnicity, which is a newly validated variable, curated from primary care and secondary care records.
4. Estimated deviation from expectation (previously framed as ‘reduction’) using a mix of two references:
   a. Projected trend based on data from Nov 2018 to Feb 2020 (14 months) – the same approach as the original submission. Unfortunately, there are no retrospective data before then
   b. An assumed flat trend from Feb 2020, adjusted for seasonal variations. This is a conservative estimate as likely that that chronic ill health and obesity levels continue to rise.
   c. Based on the two approaches, we devised a wider confidence interval of expected measurements which we believe is a more balanced approach.

There are some changes to the findings. Most notably the current estimation showed people who are older (80 or above) had much fewer measurements than expected, which largely could be due to a form of survival bias – older people who survived the Covid pandemic are likely those who had fewer long-term conditions and required fewer measurements. However, the conclusion of the paper remained largely unchanged – the observed number of BP measurements is still below expectations no matter the reference, and it is particularly worrying for younger people, especially given the importance of BP for multiple outcomes. There was also new suggestive evidence show a J-shaped pattern in deviation of BP measurements and IMD quintiles.

2) Data Availability – thank you for including a statement which requires some revision.

As the data are not freely available, please describe briefly the ethical, legal, or contractual restriction that prevents you from sharing it. Please also include an appropriate contact (web or email address) for inquiries. Please note that the point of contact cannot be a study author.

The information detailed on pages 9/10 (Ethical & data access approvals) could be placed in the submission form under the data availability sub-section.

RESPONSE: We have now revised the data availability statement to state the contractual restriction for sharing the data more widely. The statement included the BHF Data Science Centre [bhfdsc@hdruk.ac.uk](mailto:bhfdsc@hdruk.ac.uk) as a contact as a point of contact.

3) Data reporting - please ensure that the study is reported according to the STROBE guideline, and include the completed STROBE checklist as Supporting Information. Please add the following statement, or similar, to the Methods: "This study is reported as per the Strengthening the Reporting of Observational Studies in Epidemiology (STROBE) guideline (S1 Checklist)."

The STROBE guideline can be found here: https://eur03.safelinks.protection.outlook.com/?url=http%3A%2F%2Fwww.equator-network.org%2Freporting-guidelines%2Fstrobe%2F&data=05%7C02%7CFrederick.Ho%40glasgow.ac.uk%7Cc63bb58f6f464e02328808dc743959e2%7C6e725c29763a4f5081f22e254f0133c8%7C1%7C0%7C638513036865797280%7CUnknown%7CTWFpbGZsb3d8eyJWIjoiMC4wLjAwMDAiLCJQIjoiV2luMzIiLCJBTiI6Ik1haWwiLCJXVCI6Mn0%3D%7C0%7C%7C%7C&sdata=D1gFk7sfMIgYuMnzpQVASISOayY7uJTBLrZCYA6rY2Y%3D&reserved=0

When completing the checklist, please use section and paragraph numbers, rather than page and/or line numbers as these often change in the event of publication.

RESPONSE: We have now included a STROBE checklist and checked our paper is adhering to that.

4) Protocol/statistical analysis plan – did your study have a prospective protocol or analysis plan? Please state this (either way) early in the Methods section.

a) If a prospective analysis plan (from your funding proposal, IRB or other ethics committee submission, study protocol, or other planning document written before analyzing the data) was used in designing the study, please include the relevant prospectively written document with your revised manuscript as a Supporting Information file to be published alongside your study, and cite it in the Methods section. A legend for this file should be included at the end of your manuscript.

b) If no such document exists, please make sure that the Methods section transparently describes when analyses were planned, and when/why any data-driven changes to analyses took place.

c) In either case, changes in the analysis-- including those made in response to peer review comments-- should be identified as such in the Methods section of the paper, with rationale.

For all observational studies, in the manuscript text, please indicate: (1) the specific hypotheses you intended to test, (2) the analytical methods by which you planned to test them, (3) the analyses you actually performed, and (4) when reported analyses differ from those that were planned, transparent explanations for differences that affect the reliability of the study's results. If a reported analysis was performed based on an interesting but unanticipated pattern in the data, please be clear that the analysis was data-driven.

RESPONSE: We have a prospective protocol which is now included as a supplement. In addition, we have

5) Author Summary - at this stage, we ask that you include a short, non-technical Author Summary of your research to make findings accessible to a wide audience that includes both scientists and non-scientists. The authors summary should consist of 2-3 succinct bullet points under each of the following headings:

• Why Was This Study Done? Authors should reflect on what was known about the topic before the research was published and why the research was needed.

• What Did the Researchers Do and Find? Authors should briefly describe the study design that was used and the study’s major findings. Do include the headline numbers from the study, such as the sample size and key findings.

• What Do These Findings Mean? Authors should reflect on the new knowledge generated by the research and the implications for practice, research, policy, or public health. Authors should also consider how the interpretation of the study’s findings may be affected by the study limitations. In the final bullet point of ‘What Do These Findings Mean?’, please describe the main limitations of the study in non-technical language.

The Author Summary should immediately follow the Abstract in your revised manuscript. This text is subject to editorial change and should be distinct from the scientific abstract. Please see our author guidelines for more information: https://eur03.safelinks.protection.outlook.com/?url=https%3A%2F%2Fjournals.plos.org%2Fplosmedicine%2Fs%2Frevising-your-manuscript%23loc-author-summary&data=05%7C02%7CFrederick.Ho%40glasgow.ac.uk%7Cc63bb58f6f464e02328808dc743959e2%7C6e725c29763a4f5081f22e254f0133c8%7C1%7C0%7C638513036865800239%7CUnknown%7CTWFpbGZsb3d8eyJWIjoiMC4wLjAwMDAiLCJQIjoiV2luMzIiLCJBTiI6Ik1haWwiLCJXVCI6Mn0%3D%7C0%7C%7C%7C&sdata=0vSPHTZkV1vGFdcq9QFGIrcKXDGlJBPrSmTai7hxfOA%3D&reserved=0

RESPONSE: An author summary is now included.

6) Introduction – this is currently a little slender. Please ensure that you address past research and explain the need for and potential importance of your study. Indicate whether your study is novel and how you determined that. If there has been a systematic review of the evidence related to your study (or you have conducted one), please refer to and reference that review and indicate whether it supports the need for your study.

RESPONSE: We have now included additional discussions of literature in the introduction but to our knowledge there was not a lot of existing evidence.

7) Discussion - please ensure that you present and organize the Discussion as follows: a short, clear summary of the article's findings; what the study adds to existing research and where and why the results may differ from previous research; strengths and limitations of the study; implications and next steps for research, clinical practice, and/or public policy; one-paragraph conclusion.

RESPONSE: The Discussion is restructured to follow the suggestion.

# Reviewer #1

Summary: This large, population-level study used primary care data from England to describe trends in the measurement of 14 cardiometabolic risk factors from April 2019 to April 2023. It showed approximately 5.52 million fewer risk factor measurements per month than expected between March 2020 and February 2022 during pandemic restrictions, especially among those aged under 60 years. After February 2022, reductions in BMI, BP and Hba1c measurements persisted.

Originality/ importance: This study shows the power of large electronic health record datasets for analyses across a suite of cardiometabolic risk factor measures to assess pandemic impact on care. While many other studies report reductions in GP consultations for routine clinical monitoring during the pandemic, few have extended to post-pandemic time periods. There has been one large OpenSAFELY study reporting similar patterns across 11 indicators of GP clinical activity up to December 2021, accompanied by a dashboard of measures for monitoring GP clinical activity that is currently updated to December 2023. Nevertheless, the present study is a more focussed deep dive into cardiometabolic risk factor recording by age-group, sex and deprivation, with important implications for public health.

Comments

1. Check the wording of the title: an article seems to be missing

RESPONSE: We have reworded the title as ‘Impact of the COVID-19 pandemic on the decline and recovery of routine measurement of cardiometabolic disease risk factors in primary care in England’.

2. Under 'Evidence before this study', a key omission is the OpenSAFELY study of trends in GP clinical activity in England by Fisher et al(1). This should be referenced.

RESPONSE: Thank you for the suggestion, we have now included Fisher et al in the Introduction. The ‘Evidence before this study’ section is not applicable to this submission.

3. The methods section should clarify who is in the study population: was it adults only? At which timepoint(s) were they assessed for eligibility? It was also unclear why those who had died by April 2023 were excluded completely rather than being censored at death.

RESPONSE: We have now revised our analysis to include all people available.

4. The study design should be clearly stated in the methods.

RESPONSE: This is a retrospective cohort study which is now explicated stated in the paper.

5. The section describing the cardiometabolic risk factors was brief. It would be helpful to include further rationale for the choice of these risk factors and explanation about how and when they were measured.

RESPONSE: The description of the risk factors was expanded. Briefly, they included commonly used risk factors for the management and/or primary and secondary prevention of cardiometabolic disease (myocardial infarction and type 2 diabetes), and relevant complications (e.g. chronic liver and kidney diseases). We included risk factors measured were measured from Nov 2018 to March 2024.

6. Currently, the link to the pre-specified analysis plan on GitHub does not work so it is not possible to compare the pre-specified analysis plan with results presented.

RESPONSE: Apologies, there was a technical issue. We have now requested the BHF DSC administrator to enable public access to the repository which should be enabled very soon.

7. In the results section, information is needed on characteristics of the study population. The total number of individuals is given as 631 million - is this a typo?

RESPONSE: Apologies. The number was a typo and now corrected. This study included 49 million individuals.

8. In the supplementary tables, an explanatory footnote would aid interpretation of the betas.

RESPONSE: Explanatory footnotes are now added to the supplementary tables in GAMs.

7. The first sentence of the discussion is grammatically incorrect, as is the sentence in paragraph two beginning 'Regardless of the reasons.' The language of the discussion should be reviewed throughout.

RESPONSE: These two sentences in discussions are now rewritten and the paper is thoroughly reviewed for readability.

8. There was some inconsistency in interpretation of the findings related to socioeconomic status (SES): while text in the results suggested little difference in measurements by SES, except for cigarette smoking, the interpretation cautioned against virtual consultations to avoid widening inequalities among groups without access to home blood pressure monitors, especially if such deficiencies are greater in lower socioeconomic areas. However, this is not what the study found.

RESPONSE: Upon reanalysis to address editor and reviewers’ comments, we did identify a socioeconomic pattern in measurements. We have therefore rewritten the paragraph on SES.

9. In the mechanisms section, it was suggested that obesity can lead to cardiovascular disease through Hba1c. Glycated haemoglobin is a marker of blood glucose levels over the last 2-3 months and can indicate poor diabetic control, rather than a mechanism leading towards cardiovascular disease in itself. The language around this needs to be clarified.

RESPONSE: We have rewritten that paragraph, and this statement is deleted.

10. The paragraph on consistency with existing literature should mention the OpenSAFELY study and dashboard findings on routine GP clinical activity including cardiovascular risk factor measurement.

RESPONSE: The discussion with the OpenSAFELY paper is now added.

11. Further explanation is needed in the limitations about why results obtained from extrapolation of the pre-pandemic trend might be 'somewhat biased' and 'less relevant to the post-pandemic population.'

RESPONSE: We have now expanded the discussion on limitations. This is possibly due to survival effect where a proportion of people who had long term conditions have died during the Covid and the remaining population could need fewer measurements. This is speculative and other unknown factors may also operate.

References

(1). Fisher et al. Eleven key measures for monitoring routine general practice clinical activity during COVID-19: a retrospective cohort study using 48 million adults' primary care records in England through OpenSAFELY. Elife 2023; 21:e84673.

Dashboard: <https://reports.opensafely.org/reports/opensafely-sro-key-measures-dashboard/>

# Reviewer #2

This is an interesting study on the change of number of measurements of routine cardiometabolic disease risk factors in primary care following Covid-19 pandemic. However, there are a couple of major issues needing attention.

1) Counterfactual estimates. We can see from Figure 1 that the observed numbers of measurements were mostly recovered to pre-pandemic levels or following the previous trends. The key findings are in Figure 2 following the adjustment/substraction from the counterfactual estimates. Now the robustness and reliability of the counterfactual estimates become absolutely crucial in concluding that there is reduction in number of measurement. How these counterfactual estimates are exactly derived? from only these 4 years data? Normally data from longer time such as 10 to 20 years are needed to establish trends for prediction and also with all the potential predictors and risk factors. Do we have these? In other words, how reliable and robust are these counterfactual estimates?

RESPONSE: We understand the concern over the reliability of the counterfactual trends. Unfortunately, the GDPPR data are only available due to the Covid-19 pandemic and it provided two-year retrospective data from the reporting date. Therefore, about half participants had data from April 2018, and another half from October 2018. These are now described in Methods. We understand this is a strong limitation as our estimated missing measurements are based on the projected trend. Therefore, in addition to the projected trend, we also compared the observed values against another reference – a flat trend from Feb 2020. We believe this addition allows us to give a more balanced estimates of expected recovery. However, of course, it is very difficult to know what the real risk factor estimates should be based on disease burdens etc over time but at least we have given wider confidence intervals to allow readers to see patterns and where they fit within expected measurement levels.

2) In the stats analysis section, it says "Measurements of people who had died by April 2023 were excluded to eliminate competing risk". However, this is not the way to handle competing risk. The results could be biased because of this as it only applies to people alive which excluded around 200k people died in the pandemic in the UK.

RESPONSE: We have now revised our analysis to include all people available as we described in response to R1.3.

# Reviewer #3

Thank you for inviting me to review this manuscript which estimates the number of missed tests for cardiometabolic risk factors following the onset of the COVID-19 pandemic. The main finding is a significant reduction in the number of tests recorded at the onset of the pandemic, with incomplete recovery as of April 2023. These findings are a useful extension to existing literature which can help inform the continued post-pandemic recovery. However, there are a few areas of concern and some small clarifications required before recommending the manuscript for publication, highlighted below.

Major issues

* The authors provide a link to an associated GitHub repo but it is not public. This should be made public for review.

RESPONSE: Apologies, there was a technical issue. We have now made sure the GitHub is accessible.

* The codelists used to identify the various risk factor measurements are unclear. A link is provided, but this leads to the GDPPR guide for analysts. Can the authors provide a more specific link? Or highlight where they can be found in the GitHub repo?

RESPONSE: The code list is on existing clusters. We have revised the URL to ensure the correct webpage is linked.

* As the authors highlight, there was an increasing trend observed across the measurements prior to the pandemic. In some measurements, the extent of this is quite surprising. For example, there is a doubling of blood pressure measurements in the first year of the study period. This is not consistent with references 3 and 20, which show more stable rates of measurements prior to the pandemic for HbA1c, cholesterol and blood pressure. If this is not a true reflection of actual activity, it is likely the subsequent estimated number of missed measurements is overestimated. Can the authors provide any explanation for this increase?

RESPONSE: We are not certain about the inconsistency in the long-term trend being measured. We have now expanded our data for modelling from April 2019 to November 2018, as well as including the underlying population size. A similar increasing trend is still observed. We understand that long term trend based on such a short duration might not be reliable and therefore combine the projected trend with an assumed stable trend to create a wider band of expected levels. This should provide a reasonably conservative estimate assuming our population is not becoming healthier, which by all trends it is not.

- The authors describe the removal of measurements for people who have died by April 2023. Is it possible that the positive trend, at least in part, reflects an increasing denominator population as new patients can enter the study population throughout the study period? Could you instead include measurements for those that die before the end of the study period and calculate the rate with person-months as the denominator and see if the trend persists?

RESPONSE: We have now revised our analysis to include all people available as we described in response to R1.3.

Minor issues

* Title

- Include the setting

- The title suggests this study is restricted to the post-pandemic period.

- A suggested revision:

"Impact of the COVID-19 pandemic on the routine measurement of cardiometabolic disease risk factors in primary care in England"

RESPONSE: We have now revised the title as suggested with a minor change to describe the decline and recovery aspects. We hope this is acceptable.

* Abstract

- Findings. It would be useful to state the number of patients impacted by missed measurements as well as the total number of missed measurements.

RESPONSE: To address other comments on changing underlying population, we have now reported deviation per 1,000 individuals.

* Intro

- "Modelling predicted that this decline could result in an excess of over 13,000 CVD events in the Great Britain". It's not obvious this is referring to citation 2. Recommend citing again here.

RESPONSE: The reference was now added.

* Methods

- It's evident from the age groups used that this study is restricted to adults but you could more clearly state this in the text.

RESPONSE: the inclusion criteria is now made explicit in Methods.

* Results

- Including the total number of rows in GDPPR is of limited use for this study. The use of "primary care record" here could be confusing as this is more commonly used to refer to the complete set of recordings for an individual.

RESPONSE: We have now removed the total rows of GDPPR.

- You could be clearer on what the 712 million represents. This is the total number of risk factor measurements in the study population between April 2019 and April 2023.

RESPONSE: We have now revised the reporting to focus on inclusion/exclusion of individuals.

- You state that these measurements are across 631 million individuals. This is presumably missing a decimal point. If so, this is at odds with the 57 million in the Databases section, presumably due to patients meeting the age criteria during the study period, which may be worth describing.

RESPONSE: Apologies. The number was a typo and now corrected. This study included 49 million individuals.

- The order of magnitude used for the number of measurements isn't very easy to work with. It would be easier if these were presented in either thousands or millions.

RESPONSE: The presented numbers are now presented as number per 1,000.

- Table 1. Related to the above, the figure title states "number per '00,000" which suggests this is a rate.

RESPONSE: Apologies for the confusion the original presented results are in absolute numbers but now we have included results as rates (number of measurements per individuals).

- Figure 1.

- The data represented is from Apr 2019-23, but the lines appear to start before 2019. I suggest aligning the x ticks with the start of the year rather than the middle to avoid confusion.

RESPONSE: These are now corrected. In this re-analysis we included retrospective data up to November 2018.

- This could also be helped by including the date period in the figure title.

RESPONSE: Date period is added to the Figure title.

- Indicate what the shaded area represents in the legend or figure title.

RESPONSE: This is now stated in the footnote.

- Results paragraph 4 - include the absolute number as well as the percentage reduction, as in the previous paragraph.

RESPONSE: Both absolute and relative deviations are now reported.

# General editorial requests:

1. Please upload any figures associated with your paper as individual TIF or EPS files with 300dpi resolution at resubmission; please read our figure guidelines for more information on our requirements: https://eur03.safelinks.protection.outlook.com/?url=http%3A%2F%2Fjournals.plos.org%2Fplosmedicine%2Fs%2Ffigures&data=05%7C02%7CFrederick.Ho%40glasgow.ac.uk%7Cc63bb58f6f464e02328808dc743959e2%7C6e725c29763a4f5081f22e254f0133c8%7C1%7C0%7C638513036865806311%7CUnknown%7CTWFpbGZsb3d8eyJWIjoiMC4wLjAwMDAiLCJQIjoiV2luMzIiLCJBTiI6Ik1haWwiLCJXVCI6Mn0%3D%7C0%7C%7C%7C&sdata=FZd3PMj7c0ez20MCwUCc12N8lxIbtPq4dK9yZfmb3y8%3D&reserved=0. While revising your submission, please upload your figure files to the PACE digital diagnostic tool, https://eur03.safelinks.protection.outlook.com/?url=https%3A%2F%2Fpacev2.apexcovantage.com%2F&data=05%7C02%7CFrederick.Ho%40glasgow.ac.uk%7Cc63bb58f6f464e02328808dc743959e2%7C6e725c29763a4f5081f22e254f0133c8%7C1%7C0%7C638513036865810100%7CUnknown%7CTWFpbGZsb3d8eyJWIjoiMC4wLjAwMDAiLCJQIjoiV2luMzIiLCJBTiI6Ik1haWwiLCJXVCI6Mn0%3D%7C0%7C%7C%7C&sdata=QHdFr7eD0JBrEsca4q5WLfDoiRILT7f8d%2BRXUHiEYag%3D&reserved=0. PACE helps ensure that figures meet PLOS requirements. To use PACE, you must first register as a user. Then, login and navigate to the UPLOAD tab, where you will find detailed instructions on how to use the tool. If you encounter any issues or have any questions when using PACE, please email us at PLOSMedicine@plos.org.

RESPONSE: The NHS Digital does not allow the export of EPS file. We have uploaded vector PDF files as figures instead. If preferred we can convert those into raster TIFF.

2. Please ensure that the paper adheres to the PLOS Data Availability Policy (see https://eur03.safelinks.protection.outlook.com/?url=http%3A%2F%2Fjournals.plos.org%2Fplosmedicine%2Fs%2Fdata-availability&data=05%7C02%7CFrederick.Ho%40glasgow.ac.uk%7Cc63bb58f6f464e02328808dc743959e2%7C6e725c29763a4f5081f22e254f0133c8%7C1%7C0%7C638513036865813828%7CUnknown%7CTWFpbGZsb3d8eyJWIjoiMC4wLjAwMDAiLCJQIjoiV2luMzIiLCJBTiI6Ik1haWwiLCJXVCI6Mn0%3D%7C0%7C%7C%7C&sdata=kRD6m9mDUGycoKnac1NqD%2B9yB2syS8r29pV%2BCkhZzzg%3D&reserved=0), which requires that all data underlying the study's findings be provided in a repository or as Supporting Information. For data residing with a third party, authors are required to provide instructions with contact information for obtaining the data. PLOS journals do not allow statements supported by "data not shown" or "unpublished results." For such statements, authors must provide supporting data or cite public sources that include it.

RESPONSE: This is included.

3. We ask every co-author listed on the manuscript to fill in a contributing author statement, making sure to declare all competing interests. If any of the co-authors have not filled in the statement, we will remind them to do so when the paper is revised. If all statements are not completed in a timely fashion this could hold up the re-review process. If new competing interests are declared later in the revision process, this may also hold up the submission. Should there be a problem getting one of your co-authors to fill in a statement we will be in contact. YOU MUST NOT ADD OR REMOVE AUTHORS UNLESS YOU HAVE ALERTED THE EDITOR HANDLING THE MANUSCRIPT TO THE CHANGE AND THEY SPECIFICALLY HAVE AGREED TO IT. You can see our competing interests policy here: https://eur03.safelinks.protection.outlook.com/?url=http%3A%2F%2Fjournals.plos.org%2Fplosmedicine%2Fs%2Fcompeting-interests&data=05%7C02%7CFrederick.Ho%40glasgow.ac.uk%7Cc63bb58f6f464e02328808dc743959e2%7C6e725c29763a4f5081f22e254f0133c8%7C1%7C0%7C638513036865817664%7CUnknown%7CTWFpbGZsb3d8eyJWIjoiMC4wLjAwMDAiLCJQIjoiV2luMzIiLCJBTiI6Ik1haWwiLCJXVCI6Mn0%3D%7C0%7C%7C%7C&sdata=iYGJSOZKxblOj07CMVuNLcw6pi7NwxKmLK%2FVlHDnJaw%3D&reserved=0.

RESPONSE: These are declared.
