# Supplementary material for: Routine measurement of cardiometabolic disease risk factors in primary care in England before, during, and after the COVID-19 pandemic: A population-based cohort study
Source: PLoS Med. 2024 Nov 26;21(11):e1004485. doi: 10.1371/journal.pmed.1004485 (PMC11593757; doi:10.1371/journal.pmed.1004485)
Supplement: Attachment — Submitted filename: CCU008_01 number of risk factor paper r2v2 - Responses.docx [file pmed.1004485.s005.docx]

GENERAL

Thank you very much for your detailed and considered responses to previous comments. Please see below for further comments which we require that you address in full prior to publication.

Please note that some of the requests may not apply to your study design and some may have already been incorporated into the manuscript but please review the complete list and include any additional items as necessary.

COMMENTS FROM THE ACADEMIC EDITOR

I am happy to support your decision to accept the manuscript. I have two general comments that you may wish to consider when providing feedback to the authors.

First, I am not entirely convinced by the authors’ argument that pressure measurements did not return to baseline. Upon reviewing Figure 2, it appears that the measurements have largely returned to baseline, despite some periodic variability that may explain the fluctuations in confidence intervals. Additionally, I find the claim regarding fasting glucose levels not returning to baseline unpersuasive. The pre-COVID trend shows a sharp decrease, likely due to a shift towards hemoglobin A1c testing that you can kind of see in the pre-pandemic data. I believe the paper works well as a descriptive analysis, and I would encourage the authors to focus more on the broader trends and disparities related to COVID-19, rather than emphasizing the deficits in return to baseline, which the data does not seem to robustly support.

Response: Thank you for the appreciation for our study. We have reworded the description on BP as ‘prolonged recovery’ rather than ‘still under expectation’. We totally agree that the trend of fasting glucose is likely due to the accelerated shift to HbA1c, which we have discussed.

Second, while the authors appropriately identify key limitations in the discussion, I believe they could be prioritized differently. In my view, the most significant limitations are (1) the limited amount of pre-COVID data available for predictive modeling, and (2) potential data artifacts related to mortality, which may alter the denominator and introduce bias.

Response: We have rearranged the order of the limitations as suggested.

COMMENTS FROM THE EDITORS:

TITLE

Please revise your title according to PLOS Medicine's style. Your title must be nondeclarative and not a question. It should begin with main concept if possible. "Effect of" should be used only if causality can be inferred, i.e., for an RCT. Please place the study design ("A randomized controlled trial," "A retrospective study," "A modelling study," etc.) in the subtitle (ie, after a colon).

We suggest, “Routine measurement of cardiometabolic disease risk factors in primary care in England during the COVID-19 pandemic: A population-based cohort study“ or similar.

Response: We have changed the title to ‘Routine measurement of cardiometabolic disease risk factors in primary care in England before, during and after the COVID-19 pandemic: A population-based cohort study’.

DATA AVAILABILITY STATEMENT

Please also include the following details in the data availability section of the manuscript submission form when you resubmit:

* Details regarding your analysis code including the URL (and any accession codes) i.e., <https://eur03.safelinks.protection.outlook.com/?url=https%3A%2F%2Fgithub.com%2FBHFDSC%2FCCU008_01&data=05%7C02%7CFrederick.Ho%40glasgow.ac.uk%7C8f510956f925465b756308dcdbf4df07%7C6e725c29763a4f5081f22e254f0133c8%7C1%7C0%7C638627091986520839%7CUnknown%7CTWFpbGZsb3d8eyJWIjoiMC4wLjAwMDAiLCJQIjoiV2luMzIiLCJBTiI6Ik1haWwiLCJXVCI6Mn0%3D%7C0%7C%7C%7C&sdata=cXCdUGnG7UX%2BzclrzrtihYwjo09i1OYGjg%2BmqL3%2FMek%3D&reserved=0>

* A URL for e BHF Data Science Centre’s CVD-COVID-UK/COVID-IMPACT consortium and a contact email address for a Mr Rouven Priedon.

* These details from pages 9 & 10 of the PDF: ‘The data used in this study are available in NHS England’s Secure Data Environment (SDE) service for England, but as restrictions apply they are not publicly available (https://digital.nhs.uk/services/secure-data-environment-service). The CVD-COVID-UK/COVID-IMPACT programme led by the BHF Data Science Centre (https://bhfdatasciencecentre.org/) received approval to access data in NHS England’s SDE service for England from the Independent Group Advising on the Release of Data (IGARD) (https://digital.nhs.uk/about-nhs-digital/corporate-information-and-documents/independent-group-advising-on-the-release-of-data) via an application made in the Data Access Request Service (DARS) Online system (ref. DARS-NIC-381078-Y9C5K) (https://digital.nhs.uk/services/data-access-request-service-dars/dars-products-and-services). The CVD-COVID-UK/COVID-IMPACT Approvals & Oversight Board (https://bhfdatasciencecentre.org/areas/cvd-COVID-uk-COVID-impact/) subsequently granted approval to this project to access the data within NHS England’s SDE service for England. The de-identified data used in this study were made available to accredited researchers only as per the data sharing agreement and the ethical approval. Those wishing to gain access to the data should contact Mr Rouven Priedon (bhfdsc@hdruk.ac.uk) in the first instance.’

Response: These are now included in the submission system.

ABSTRACT

Please structure your abstract using the PLOS Medicine headings (Background, Methods and Findings, Conclusions).

Please combine the Methods and Findings sections into one section, “Methods and findings”.

Response: This is now changed.

Abstract Methods and Findings:

Please ensure that all numbers presented in the abstract are present and identical to numbers presented in the main manuscript text.

Response: These were checked to be consistent.

Please include the study design, population and setting, number of participants, years during which the study took place, length of follow up, and main outcome measures.

Response: These are all included.

Please quantify the main results (with 95% CIs and p values).

Please include the important dependent variables that are adjusted for in the analyses.

Please include the actual amounts and/or absolute risk(s) of relevant outcomes (including NNT or NNH where appropriate), not just relative risks or correlation coefficients. (example for absolute risks: PMID: 28399126).

Please include a summary of adverse events if these were assessed in the study.

In the last sentence of the Abstract Methods and Findings section, please describe the main limitation(s) of the study's methodology.

Response: Not applicable.

AUTHOR SUMMARY

Thank you for including an author summary which reads very nicely. Please see below for some minor suggestions:

* Why Was This Study Done?

Bullet point #2 suggest: ‘However, the extent to which recovery has occurred throughout 2024, remains unknown as do outcomes according to age, sex, ethnicity or deprivation.’ Or similar

Response: Thank you for the suggestion. This is slightly reworded as ‘•	However, the extent to which recovery has occurred until 2024, and how recovery vary by age, sex, ethnicity or deprivation, remains unknown’

* What Did the Researchers Do and Find?

Bullet point #1: suggest, ‘…which covers…’

Bullet point #2: suggest, ‘…risk factor…’

Response: These are changed as suggested.

* What Do These Findings Mean?

Please include a final bullet point of ‘What Do These Findings Mean?’, please describe the main limitations of the study in non-technical language.

Response: These are now added.

METHODS and RESULTS

Please note the comments above from the academic editor.

Response: We have toned down the emphasis on BP measurements under expectation and reworded as ‘prolonged recovery’. We have removed mentioning of fasting glucose in the first paragraph of discussion.

Please move the ethics statement from page 9 and include in the methods section.

Response: This is moved to the end of the Methods section.

TABLES and FIGURES

Throughout the main manuscript, please name figure labels using Arabic numerals, and abbreviate the word “Figure” to “Fig” (e.g., Fig 1, Fig 2, Fig 3, etc.

Please Cite figures with the format: Fig 1, Fig 2, Fig 3, etc.

Response: These are renamed.

Please ensure that all abbreviations are defined in the caption or an appropriate footnote, including those used to report statistical information.

Please ensure to include the meaning of any dots/lines/bars.

Response: These are added.

Please consider avoiding the use of red and green in order to make figures more accessible to those with colour blindness.

Response: The Fig 3 was checked against for several common types of colour blindness and should be readable.

To help facilitate transparent data reporting, where adjusted analyses are presented please also present the unadjusted analyses for comparison. In a caption or footnote please detail all factors adjusted for.

Response: Not applicable.

DISCUSSION

Please amend the sub-heading to read ‘Discussion’ (as opposed to Discussions).

Response: This is now changed.

Please remove all information on page 9/10 except the acknowledgements and include only in the relevant sections of the manuscript submission form and methods as detailed above (DATA AVAILABILITY STATEMENT).

Response: These are now deleted.

REFERENCES

For in-text reference callouts, please place citations in square as opposed to semi-circular brackets.

Response: Square brackets are now used.

SUPPORTING INFORMATION

In the published article, supporting information files are accessed only through a hyperlink attached to the captions. For this reason, you must list captions at the end of your manuscript file. You may include a caption within the supporting information file itself, as long as that caption is also provided in the manuscript file. Do not submit a separate caption file.

Response: This is listed before the References.

When supplementary files are contained with a single file:

Please label the file as ‘S1 Supporting Information’.

Response: Two files are uploaded: one as a Word (S1) document containing all figures; the other (S2) as an Excel document containing all tables (which are too big to be included in the Word document).

Please apply alphabetical labelling to each table and figure contained within the S1 file. For example, ‘Fig A’ to ‘Fig Z’ and ‘Table A’ to ‘Table Z’.

Response: These are renamed.

Plain text does not need to be labelled and can just be given a title as necessary. For example, ‘Statistical Analysis Plan’.

Response: Not applicable.

Please cite tables/figures as ‘Fig A in S1 Supporting Information’ and/or ‘Table A in S1 Supporting Information’, for example.

Response: These are now changed as required.

Please cite plain text as, ‘Statistical Analysis Plan in S1 Supporting Information’, for example.

Response: Not applicable.

Alternatively when supplementary files are uploaded as separate files:

Please label tables as ‘S1 Table’ (so on) Please label figures as ‘S1 Fig’ (and so on) Any additional documents (protocols/analysis plans etc.) can be labelled as ‘S1 Protocol’, for example.

Please cite items as exactly as labelled.

Response: Not applicable.

STROBE Checklist – thank you for including the checklist. Please amend to refer to section and paragraph numbers as opposed to page (or line) numbers as the latter often change at the time of publication.

Response: Checklist is updated.

SOCIAL MEDIA

To help us extend the reach of your research, please detail any X (formerly Twitter) handles you wish to be included when we tweet this paper (including your own, your coauthors’, your institution, funder, or lab) in the manuscript submission form when you re-submit the manuscript.

Response: Ok.
